# Response to First‐Line Chemotherapy Predicts Response to Maintenance Avelumab Therapy in Japanese Patients With Advanced Urothelial Carcinoma

**DOI:** 10.1111/iju.70162

**Published:** 2025-07-02

**Authors:** Satoshi Inoue, Akira Hayakawa, Mikinori Kobayashi, Yuriko Nagasaka, Takanao Omi, Noritoshi Shamoto, Fumihiro Ito, Takuma Yuba, Yuri Yuguchi, Hideji Kawanishi, Kosuke Tochigi, Shusuke Akamatsu

**Affiliations:** ^1^ Department of Urology Nagoya University Graduate School of Medicine Nagoya Japan; ^2^ Department of Urology Yokkaichi Municipal Hospital Yokkaichi Japan; ^3^ Department of Urology Japanese Red Cross Aichi Medical Center Nagoya Daiichi Hospital Nagoya Japan; ^4^ Department of Urology Japanese Red Cross Aichi Medical Center Nagoya Daini Hospital Nagoya Japan; ^5^ Department of Urology Toyohashi Municipal Hospital Toyohashi Japan; ^6^ Department of Urology Chukyo Hospital Nagoya Japan; ^7^ Department of Urology Gifu Prefectural Tajimi Hospital Tajimi Japan; ^8^ Department of Urology Kariya Toyota General Hospital Kariya Japan; ^9^ Department of Urology Komaki City Hospital Komaki Japan; ^10^ Department of Urology Nagoya Medical Center Nagoya Japan

**Keywords:** avelumab, carcinoma, immunotherapy, prognosis, risk factors

## Abstract

**Objectives:**

The association between the response to first‐line chemotherapy and maintenance of avelumab remains unclear. We identified factors associated with the response to avelumab in patients with advanced urothelial carcinoma using real‐world data.

**Methods:**

We retrospectively enrolled 100 patients with advanced urothelial carcinoma treated with maintenance avelumab therapy between March 2021 and April 2024 at Nagoya University and nine affiliated hospitals. The complete/partial‐response group was defined as patients with complete response or partial response as the best response to first‐line chemotherapy. The stable disease group was defined as patients with stable disease as the best response to first‐line chemotherapy.

**Results:**

Seven patients (7.0%) achieved complete response, 65 (65.0%) achieved partial response, and 28 (28.0%) achieved stable disease as the best response to first‐line chemotherapy. Regarding avelumab therapy, the complete/partial‐response group had significantly better progression‐free survival than the stable disease group (median: 11.1 vs. 3.2 months, *p* < 0.001). In multivariate analyses, the best response to first‐line chemotherapy was the only independent risk factor for progression‐free survival (hazard ratio = 1.844, 95% confidence interval = 1.002–3.394; *p* = 0.049). Overall survival was significantly shorter in the stable disease group than in the complete/partial‐response group (median: 14.1 months vs. not reached, *p* < 0.001). Multivariate analyses revealed significant associations between poor overall survival and performance status (hazard ratio = 2.175, 95% confidence interval = 1.030–4.592; *p* = 0.042) and the best response to first‐line chemotherapy (hazard ratio = 4.174, 95% confidence interval = 1.975–8.824; *p* < 0.001).

**Conclusions:**

The best response to first‐line chemotherapy may predict the clinical outcome of patients with advanced urothelial carcinoma treated with avelumab.

**Abbreviations**
CI: confidence interval
CR: complete response
ECOG‐PS: Eastern Cooperative Oncology Group performance status
EV: enfortumab vedotin
GC: gemcitabine and cisplatin
HR: hazard ratio
ICI: immune checkpoint inhibitor
irAEs: immune‐related adverse events
L: first‐line
MHC: major histocompatibility complex
NLR: neutrophil‐to‐lymphocyte ratio
ORR: overall response rate
OS: overall survival
PD: progressive disease
PD‐1: programmed death 1
PD‐L1: programmed death ligand 1
PFS: progression‐free survival
PR: partial response
RECIST: Response Evaluation Criteria in Solid Tumors
SD: stable disease
UC: urothelial carcinoma

## 1 Introduction

Historically, platinum‐based combination chemotherapy has been the standard first‐line (1 L) therapy for advanced or metastatic urothelial carcinoma (UC); however, most patients progress within approximately 9 months [1]. As post‐treatment for platinum‐based combination chemotherapy, the approval of anti‐programmed death ligand 1 (PD‐L1) and anti‐programmed death 1 (PD‐1) agents provides additional treatment options for patients with metastatic UC. In the phase 3 JAVELIN Bladder 100 trial, 1 L maintenance with avelumab (anti‐PD‐L1) plus best supportive care significantly prolonged overall survival (OS) compared with best supportive care alone in patients with metastatic UC without progression after 1 L platinum‐based chemotherapy [2]. This switch to maintenance avelumab therapy was approved by the Japanese Pharmaceuticals and Medical Devices Agency in January 2021.

Recently, a combination of enfortumab vedotin (EV), an antibody‐drug conjugate targeting Nectin‐4, and pembrolizumab was approved as a 1 L therapy for advanced UC [3]. A meta‐analysis showed that although the OS of EV and pembrolizumab combination therapy is significantly longer than that of platinum‐based chemotherapy, the incidence of serious adverse reactions to EV and pembrolizumab is significantly higher than that to platinum‐based chemotherapy [4]. Therefore, the appropriate selection of therapeutic agents is necessary for patients with advanced UC.

Bellmunt et al. [5] reported that an Eastern Cooperative Oncology Group performance status (ECOG‐PS) > 0, anemia, and liver metastasis predict outcomes in patients with metastatic UC receiving second‐line chemotherapy. Recently, various risk factors, such as the Bellmunt prognostic risk factors, have been reported to be associated with clinical outcomes in patients with metastatic UC treated with pembrolizumab, a PD‐1 inhibitor [6]. Moreover, the pembrolizumab response rate is much higher in 1 L chemotherapy responders than in 1 L chemotherapy nonresponders [7]. However, to the best of our knowledge, the relationship between the best response to 1 L chemotherapy and efficacy of maintenance avelumab therapy in patients with advanced UC has not been investigated. Therefore, the aim of this study was to evaluate the prognostic impact of the best response to 1 L chemotherapy in patients with advanced UC treated with maintenance avelumab therapy.

## 2 Methods

### 2.1 Patient Selection

This retrospective multicenter collaborative study was conducted as part of the overarching MEGUMI (MEidai GenitoUrinary Mega Investigation) project. This study conformed to the provisions of the Declaration of Helsinki and current ethical guidelines. The entire study was centrally approved by the institutional review board of Nagoya University Graduate School of Medicine (approval number: 2016‐0474‐5) and all hospitals included in this study. The requirement for written informed consent from the participants was waived owing to the retrospective nature of the study.

We registered 100 patients who were treated with maintenance avelumab therapy for advanced UC between February 2021 and April 2024 at Nagoya University and nine other affiliated hospitals in Japan. All patients received intravenous avelumab at a dose of 10 mg/kg every 2 weeks after platinum‐based chemotherapy. Treatment generally continued until tumor progression or intolerable adverse effects occurred. The following clinical and pathological data of 100 patients were retrospectively collected: age, sex, ECOG‐PS, histological subtype, primary tumor site, hydronephrosis, smoking history, resection of the primary lesion, perioperative systemic chemotherapy, hemoglobin concentration, neutrophil‐to‐lymphocyte ratio (NLR), metastatic sites, 1 L chemotherapy regimen, cycles of 1 L chemotherapy, and immune‐related adverse events (irAEs). Blood test data were collected within 2 weeks before the start of avelumab treatment. The NLR was defined as the absolute neutrophil count divided by the absolute lymphocyte count. Pathological analysis results, including tumor node metastasis stage, were categorized according to the classification system in the eighth edition of the American Joint Committee on Cancer/TNM staging system. IrAEs were defined as AEs having a potential immunological basis that required more frequent monitoring and potential intervention and graded according to the Common Terminology Criteria for Adverse Events version 5.0 [8]. The response rates of patients undergoing 1 L chemotherapy and maintenance avelumab therapy were assessed according to the Response Evaluation Criteria in Solid Tumors (RECIST) version 1.1 [9]. The definition of “best response rate” was the rate at which the size of the target lesion was maximally reduced. The CR/PR group was defined as patients with a complete response (CR) or partial response (PR) as the best response to 1 L chemotherapy, and the SD group was defined as patients with stable disease (SD) as the best response to 1 L chemotherapy. The patients were divided into two groups: the CR/PR group and the SD group.

### 2.2 Statistical Analysis

OS was defined as the time from the start of maintenance avelumab therapy to death from any cause or the last follow‐up. Progression‐free survival (PFS) was defined as the time from the start of maintenance avelumab therapy to any clinical or radiological disease progression or the last follow‐up. The cut‐off value of the NLR was set to 3, based on a previous study [10].

Continuous variables are reported as medians with ranges and were compared using the Mann–Whitney *U* test. Categorical variables were compared using the Fisher exact test. Univariate and multivariate analyses were performed using the Cox proportional hazards model. Survival analyses, including PFS and OS, were performed using Kaplan–Meier curves and compared using log‐rank tests. All statistical analyses were performed using GraphPad Prism 5 (GraphPad Software, San Diego, CA, USA) and the freely available and easy‐to‐use EZR software (Easy R, Saitama Medical Center, Jichi Medical University, Saitama, Japan), which is a graphical user interface for R (R Foundation for Statistical Computing, Vienna, Austria) [11]. *p* values were two‐sided, and statistical significance was set at *p* < 0.05.

## 3 Results

### 3.1 Baseline Clinicopathological Features

The baseline clinicopathological characteristics of the patients are summarized in Table 1. The median age of the patients treated with avelumab was 74 years (range 43–87 years). Among the 100 patients, 79 (79.0%) were men and 30 (30.0%) were nonsmokers. The ECOG‐PS score was 0 in 66 (66.0%), 1 in 28 (28.0%), and ≥ 2 in 5 (5.0%) patients. The primary tumor site of UC was the upper urinary tract in 34 (34.0%), bladder in 61 (61.0%), and both in 5 (5.0%) patients. A hemoglobin concentration of < 10 g/dL was observed in 39 patients (39.0%). Liver metastases were detected in 10 (10.0%) patients. Fifty‐seven patients (57.0%) underwent primary lesion resection. Forty‐nine (49.0%) patients received gemcitabine and cisplatin (GC) as 1 L chemotherapy, and the number of 1 L chemotherapy cycles was 4 in 69 (69.0%) patients. Thirty‐four (34.0%) patients experienced irAEs. The details of the irAEs are described in Table S1. Of the 100 patients, 7 (7.0%) achieved CR, 65 (65.0%) achieved PR, and 28 (28.0%) showed SD at the point of maximum effect of 1 L chemotherapy as assessed by RECIST.

**TABLE 1:** Clinicopathological characteristics of patients classified by the best response of first‐line chemotherapy.

Variables	Total	First‐line chemotherapy		*p*
		The CR/PR	The SD	
	*n* = 100	*n* = 72	*n* = 28	
Age (year)				
Median	74	74	73	0.095
Range	43–87	49–87	43–85	
Follow‐up duration (month)				
Median	12.5	14.7	10.1	0.013
Range	2.0–37.8	2.1–37.8	2.0–30.1	
Cycles of avelumab				
Median	11	13	5.5	0.003
Range	1–59	1–59	2–26	
Sex, *n* (%)				
Male	79 (79.0)	56 (77.8)	23 (82.1)	0.787
Female	21 (21.0)	16 (22.2)	5 (17.9)	
ECOG‐PS, *n* (%)				
0	66 (66.0)	49 (68.1)	17 (60.7)	0.050
1	28 (28.0)	21 (27.8)	7 (25.0)	
2–4	5 (5.0)	1 (1.4)	4 (14.3)	
Unknown	1 (1.0)	1 (1.4)	0 (0.0)	
Histology, *n* (%)				
Pure UC	91 (91.0)	66 (91.7)	25 (89.3)	0.707
UC with divergent differentiation or histological subtype	9 (9.0)	6 (8.3)	3 (10.7)	
Primary site, *n* (%)				
Upper urinary tract	34 (34.0)	22 (30.6)	12 (42.9)	0.551
Bladder	61 (61.0)	46 (63.9)	15 (53.6)	
Both	5 (5.0)	4 (5.6)	1 (3.6)	
Hydronephrosis, *n* (%)				
Yes	33 (33.0)	21 (29.2)	12 (42.9)	0.244
No	65 (65.0)	49 (68.1)	16 (57.1)	
Unknown	2 (2.0)	2 (2.8)	0 (0.0)	
Smoking status, *n* (%)				
Never smoker	30 (30.0)	24 (33.3)	6 (21.4)	0.342
Former smoker	45 (45.0)	31 (43.1)	14 (50.0)	
Current smoker	18 (18.0)	11 (15.3)	7 (25.0)	
Unknown	7 (7.0)	6 (8.3)	1 (3.6)	
Metastatic or locally advanced disease, *n* (%)				
Metastatic	97 (97.0)	70 (97.2)	27 (96.4)	1.000
Locally advanced	3 (3.0)	2 (2.8)	1 (3.6)	
Surgical removal of primary organ, *n* (%)				
Yes	57 (57.0)	41 (56.9)	16 (57.1)	1.000
No	43 (43.0)	31 (43.1)	12 (42.9)	
Neoadjuvant chemotherapy, *n* (%)				
Yes	19 (19.0)	11 (15.3)	8 (28.6)	0.158
No	81 (81.0)	61 (84.7)	20 (71.4)	
Adjuvant chemotherapy, *n* (%)				
Yes	10 (10.0)	5 (6.9)	5 (17.9)	0.137
No	90 (90.0)	67 (93.1)	23 (82.1)	
Hemoglobin concentration < 10 g/dL, *n* (%)				
Yes	39 (39.0)	27 (37.5)	12 (42.9)	0.653
No	61 (61.0)	45 (62.5)	16 (57.1)	
NLR > 3, *n* (%)				
Yes	38 (38.0)	27 (37.5)	11 (39.3)	0.812
No	56 (56.0)	42 (58.3)	14 (50.0)	
Unknown	6 (6.0)	3 (4.2)	3 (10.7)	
Lymph node‐only metastasis, *n* (%)				
Yes	37 (37.0)	29 (40.3)	8 (28.6)	0.358
No	63 (63.0)	43 (59.7)	20 (71.4)	
Visceral metastasis, *n* (%)				
Yes	54 (54.0)	36 (50.0)	18 (64.3)	0.265
No	46 (46.0)	36 (50.0)	10 (35.7)	
Liver metastasis, *n* (%)				
Yes	10 (10.0)	4 (5.6)	6 (21.4)	0.027
No	90 (90.0)	68 (94.4)	22 (78.6)	
Bellmunt risk factors^a^, *n* (%)				
0	44 (44.0)	34 (47.2)	10 (35.7)	0.471
1	33 (33.0)	24 (33.3)	9 (32.1)	
2	17 (17.0)	10 (13.9)	7 (25.0)	
3	5 (5.0)	3 (4.2)	2 (7.1)	
Unknown	1 (1.0)	1 (1.4)	0 (0.0)	
First‐line chemotherapy regimen, *n* (%)				
GC	49 (49.0)	34 (47.2)	15 (53.6)	0.630
G‐Carbo	41 (41.0)	31 (43.1)	10 (35.7)	
ddMVAC	6 (6.0)	5 (6.9)	1 (3.6)	
GC or G‐Carbo^b^	4 (4.0)	2 (2.8)	2 (7.1)	
Cycles of first‐line chemotherapy, *n* (%)				
< 4	17 (17.0)	10 (13.9)	7 (25.0)	0.295
4	69 (69.0)	50 (69.4)	19 (67.9)	
5–6	14 (14.0)	12 (16.7)	2 (7.1)	
Subsequent anticancer therapy, *n* (%)				
Platinum‐based	7 (7.0)	7 (9.7)	0 (0.0)	0.062
Pembrolizumab	2 (2.0)	1 (1.4)	1 (3.6)	
Enfortumab vedotin	36 (36.0)	25 (34.7)	11 (39.3)	
Taxane‐based	2 (2.0)	0 (0.0)	2 (7.1)	
Best supportive care	26 (26.0)	17 (23.6)	9 (32.1)	
Continuing avelumab	27 (27.0)	22 (30.6)	5 (17.9)	
IrAE, *n* (%)				
Yes	34 (34.0)	28 (38.9)	6 (21.4)	0.108
No	66 (66.0)	44 (61.1)	22 (78.6)	

Abbreviations: CR, complete response; ddMVAC, dose‐dense methotrexate, vinblastine, doxorubicin, and cisplatin; ECOG‐PS, Eastern Cooperative Oncology Group performance status; G‐Carbo, Gemcitabine and carboplatin; GC, gemcitabine and cisplatin; irAEs, immune‐related adverse events; NLR, neutrophil‐to‐lymphocyte ratio; PR, partial response; SD, stable disease; UC, urothelial carcinoma.

^a^ Bellmunt risk factors include an ECOG‐PS score > 0, a hemoglobin concentration of < 10 g/dL, and the presence of liver metastases.

^b^ This category includes patients who switched platinum‐based regimens while receiving first‐line chemotherapy.

Among the 100 patients, 72 (72.0%) were in the CR/PR group and 28 (28.0%) were in the SD group. The relationship between the best response to 1 L chemotherapy and clinicopathological characteristics is shown in Table 1. Liver metastasis was significantly different between the CR/PR group and the SD group (*p* = 0.027).

### 3.2 Clinical Responses to 1 L Chemotherapy and Avelumab Therapy

**FIGURE 1:**
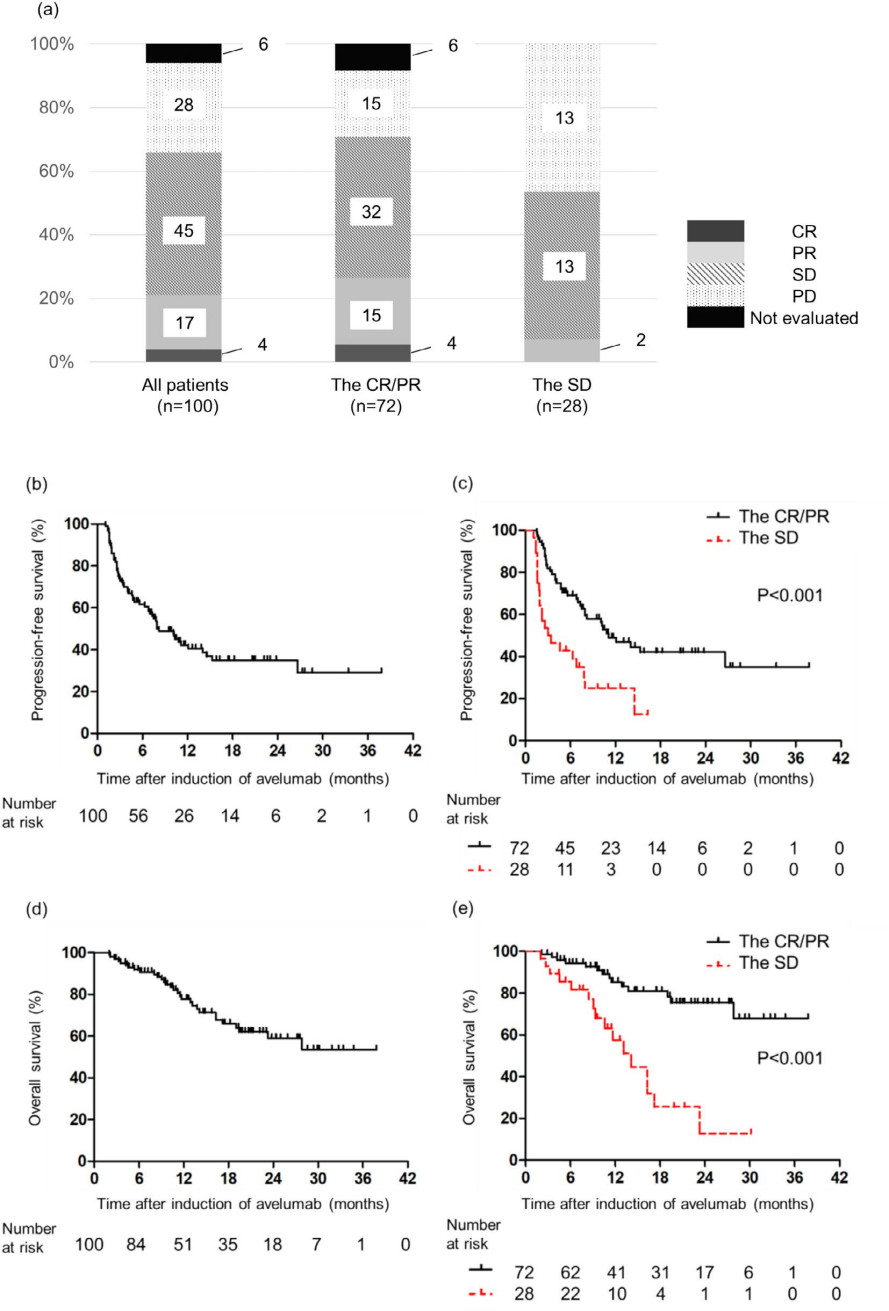
Clinical responses to first‐line (1 L) chemotherapy and avelumab treatment. (a) Frequency of best overall response to avelumab in all patients, the CR/PR group, and the SD group. Kaplan–Meier curve for time to progression‐free survival (PFS) and overall survival (OS) in patients. (b) PFS of all patients. (c) PFS of all patients according to the best response to first‐line (1 L) chemotherapy. (d) OS of all patients. (e) OS of all patients according to the best response to 1 L chemotherapy. CR, complete response; PD, progressive disease; PR, partial response; SD, stable disease.

The best responses to avelumab therapy were CR, PR, SD, and progressive disease (PD), and not evaluable in 4 (4.0%), 17 (17.0%), 45 (45.0%), 28 (28.0%), and 6 (6.0%) patients, respectively. According to RECIST, the overall response rate (ORR) and disease control rate were 21.0% and 66.0%, respectively. The CR/PR group tended to have a higher ORR to avelumab therapy (26.4% vs. 7.1%; *p* = 0.053) and disease control rate (70.8% vs. 53.6%; *p* = 0.157) than the SD group (Figure 1a).

The median PFS from the initiation of maintenance avelumab therapy in the entire cohort was 8.2 months (95% confidence interval [CI], 6.8–14.0) (Figure 1b). A Kaplan–Meier curve revealed that PFS was significantly longer in the CR/PR group than in the SD group (*p* < 0.001) (Figure 1c). Univariate Cox regression analysis showed that ECOG‐PS (*p* = 0.001), NLR (*p* = 0.038), lymph node‐only metastasis (*p* = 0.041), visceral metastasis (*p* = 0.015), liver metastasis (*p* = 0.001), irAE (*p* = 0.024) and best response to 1 L chemotherapy (*p* < 0.001) were significant risk factors for PFS. In the multivariate analysis, the best response to 1 L chemotherapy was the only independent risk factor for PFS (hazard ratio [HR] = 1.844, 95% CI = 1.002–3.394; *p* = 0.049) (Table 2).

**TABLE 2:** Univariate and multivariate Cox regression analyses influencing PFS in all patients.

Variables (comparator vs. reference)	PFS			
	Univariate		Multivariate^a^	
	HR (95% CI)	*p*	HR (95% CI)	*p*
Sex (male vs. female)	1.001 (0.539–1.857)	0.998		
ECOG‐PS (≧ 1 vs. 0)	2.351 (1.396–3.959)	0.001	1.745 (0.949–3.207)	0.073
UC with divergent differentiation or histological subtype vs. pure UC	0.482 (0.151–1.540)	0.218		
Primary tumor site (bladder vs. upper tract)	0.869 (0.502–1.507)	0.618		
Hydronephrosis (yes vs. no)	1.278 (0.744–2.198)	0.374		
Smoking (yes vs. no)	0.972 (0.552–1.709)	0.921		
Locally advanced vs. metastatic	0.936 (0.228–3.840)	0.927		
Surgical removal of primary organ (yes vs. no)	0.865 (0.516–1.450)	0.582		
Neoadjuvant chemotherapy	0.938 (0.497–1.770)	0.843		
Adjuvant chemotherapy	0.525 (0.190–1.451)	0.214		
Hemoglobin concentration < 10 g/dL (yes vs. no)	1.471 (0.879–2.461)	0.142		
NLR > 3 (yes vs. no)	1.787 (1.033–3.094)	0.038	1.382 (0.760–2.513)	0.289
Lymph node‐only metastasis (yes vs. no)	0.560 (0.321–0.978)	0.041	0.777 (0.277–2.178)	0.632
Visceral metastasis (yes vs. no)	1.931 (1.139–3.275)	0.015	1.386 (0.529–3.633)	0.506
Liver metastasis (yes vs. no)	3.579 (1.649–7.769)	0.001	1.351 (0.523–3.491)	0.535
First‐line chemotherapy regimen (G‐Carbo vs. GC)	1.188 (0.692–2.038)	0.532		
Cycles of first‐line chemotherapy (5–6 vs. 4)	1.645 (0.836–3.236)	0.149		
IrAE (yes vs. no)	0.508 (0.282–0.915)	0.024	0.596 (0.315–1.129)	0.112
Best response to first‐line chemotherapy (SD vs. CR/PR)	2.530 (1.469–4.357)	< 0.001	1.844 (1.002–3.394)	0.049

Abbreviations: CI, confidence interval; CR, complete response; ECOG‐PS, Eastern Cooperative Oncology Group performance status; G‐Carbo, Gemcitabine and carboplatin; GC, gemcitabine and cisplatin; HR, hazard ratio; irAEs, immune‐related adverse events; NLR, neutrophil‐to‐lymphocyte ratio; PFS, progression‐free survival; PR, partial response; SD, stable disease; UC, urothelial carcinoma.

^a^ Data for each parameter with *p* < 0.05 in univariate analysis is shown.

The median OS from the initiation of maintenance avelumab therapy in the whole cohort had not yet been reached, and the 1‐ and 3‐year OS rates in the whole cohort were 77.7% and 53.6%, respectively (Figure 1d). The CR/PR group had a longer OS than the SD group (*p* < 0.001) (Figure 1e). Univariate analysis revealed that ECOG‐PS (*p* = 0.019), liver metastasis (*p* = 0.028), and best response to 1 L chemotherapy (*p* < 0.001) were significant clinical risk factors for OS. Multivariate analysis revealed significant associations between poor OS and ECOG‐PS (HR = 2.175, 95% CI = 1.030–4.592; *p* = 0.042) and best response to 1 L chemotherapy (HR = 4.174, 95% CI = 1.975–8.824; *p* < 0.001) (Table 3).

**TABLE 3:** Univariate and multivariate Cox regression analyses influencing OS in all patients.

Variables (comparator vs. reference)	OS			
	Univariate		Multivariate^a^	
	HR (95% CI)	*p*	HR (95% CI)	*p*
Sex (male vs. female)	1.546 (0.591–4.042)	0.374		
ECOG‐PS (≧ 1 vs. 0)	2.355 (1.150–4.824)	0.019	2.175 (1.030–4.592)	0.042
UC with divergent differentiation or histological subtype vs. pure UC	0.423 (0.058–3.113)	0.398		
Primary tumor site (bladder vs. upper tract)	0.636 (0.305–1.325)	0.227		
Hydronephrosis (yes vs. no)	0.891 (0.408–1.947)	0.772		
Smoking (yes vs. no)	0.737 (0.347–1.562)	0.425		
Locally advanced vs. metastatic	0.714 (0.097–5.263)	0.741		
Surgical removal of primary organ (yes vs. no)	0.772 (0.377–1.583)	0.481		
Neoadjuvant chemotherapy	0.598 (0.209–1.714)	0.339		
Adjuvant chemotherapy	1.102 (0.332–3.655)	0.873		
Hemoglobin concentration < 10 g/dL (yes vs. no)	1.680 (0.821–3.440)	0.156		
NLR > 3 (yes vs. no)	1.763 (0.827–3.761)	0.142		
Lymph node‐only metastasis (yes vs. no)	0.923 (0.439–1.942)	0.833		
Visceral metastasis (yes vs. no)	1.568 (0.753–3.267)	0.230		
Liver metastasis (yes vs. no)	2.969 (1.124–7.840)	0.028	1.426 (0.509–3.996)	0.500
First‐line chemotherapy regimen (G‐Carbo vs. GC)	0.907 (0.429–1.919)	0.799		
Cycles of first‐line chemotherapy (5–6 vs. 4)	1.301 (0.480–3.523)	0.605		
IrAE (yes vs. no)	0.726 (0.332–1.588)	0.423		
Best response to first‐line chemotherapy (SD vs. CR/PR)	4.494 (2.167–9.320)	< 0.001	4.174 (1.975–8.824)	< 0.001

Abbreviations: CI, confidence interval; CR, complete response; ECOG‐PS, Eastern Cooperative Oncology Group performance status; G‐Carbo, Gemcitabine and carboplatin; GC, gemcitabine and cisplatin; HR, hazard ratio; irAEs, immune‐related adverse events; NLR, neutrophil‐to‐lymphocyte ratio; OS, overall survival; PR, partial response; SD, stable disease; UC, urothelial carcinoma.

^a^ Data for each parameter with *p* < 0.05 in univariate analysis is shown.

Next, we evaluated the effect of avelumab in the PR and SD groups with residual tumors. PFS and OS were significantly prolonged in the PR group compared to the SD group (*p* < 0.001 and *p* < 0.001, respectively) (Figure S1).

### 3.3 Conditional Survival in Patients Treated for ≥ 1 Year

**FIGURE 2:**
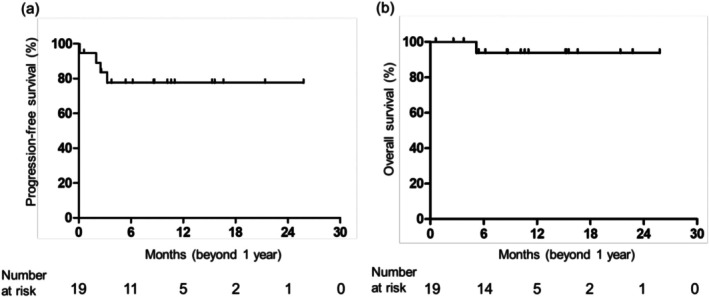
Kaplan–Meier curve for time to additional progression‐free survival (a) and additional overall survival (b) in patients who received 1 year of avelumab.

This study focused on patients who had been treated with avelumab for ≥ 1 year. The baseline clinicopathological characteristics of the patients are summarized in Table S2. The median number of avelumab cycles was 37 (range 19–59). Among the 100 patients, 19 (19.0%) were treated with avelumab for ≥ 1 year, none of whom presented with liver metastasis. The ECOG‐PS score was 0 in 14 (73.7%) patients and 1 in 5 (26.3%) patients. Among the 19 patients, 17 (89.5%) were in the CR/PR group, and two (10.5%) were in the SD group. In patients who received avelumab therapy for 1 year, the probabilities of an additional 1 year of PFS and OS were 77.6% and 93.8%, respectively (Figure 2). Next, we evaluated the effect of subsequent anticancer drug therapy on patients discontinuing avelumab. In the 100 patients, subsequent anticancer drug therapy was received by 47 patients (47.0%), including EV therapy in 36 patients (36.0%) (Table 1). The median OS from the initiation of maintenance avelumab therapy in the patients who received EV therapy was 27.8 months (95% CI, 13.8–not available) (Figure S2).

## 4 Discussion

This study evaluated the prognostic value of clinical characteristics, including the best response to 1 L chemotherapy and maintenance avelumab therapy, in patients with advanced UC. The results showed that the best response to 1 L chemotherapy was associated with the response to maintenance avelumab therapy in patients with advanced UC.

Chemotherapy destroys tumor cells through direct cytotoxic effects and promotes antitumor immune responses by inducing immunogenic cell death [12]. Gemcitabine suppresses B‐cell proliferation and antibody production in response to tumor antigens, a phenomenon that may bias antitumor immunity toward beneficial T‐cell responses [13]. Gemcitabine‐induced apoptosis in established tumors may promote the dendritic cell‐dependent cross‐presentation of tumor antigens to T cells. Platinum‐based chemotherapies, including cisplatin and carboplatin, play an important role in the antitumor immune response by inducing immunogenic cell death [14]. The mechanisms of antitumor immunomodulation by cisplatin also include upregulation of major histocompatibility complex (MHC) class I expression, recruitment and proliferation of effector cells, upregulation of the lytic activity of cytotoxic effectors, and downregulation of the immunosuppressive microenvironment [15, 16]. Cisplatin stimulates T‐cell function and enhances tumor antigen presentation by upregulating MHC class I expression [16].

Platinum‐based anticancer drugs can induce immunomodulatory effects, thereby enhancing concomitant PD‐L1 and PD‐1 blockade [17]. Cisplatin increases the cell surface levels of PD‐L1 and MHC class I molecules in several cell lines [18]. In non‐small cell lung cancer, cisplatin enhances PD‐L1 expression early and sustainably in preclinical models and patient cohorts [19]. In ovarian cancer cells, a better response to cisplatin modifies the tumor microenvironment in favor of immunogenic tumor cell death, resulting in antitumor immunity [20]. Therefore, this study suggested that the response to 1 L chemotherapy was associated with the efficacy of avelumab.

In patients with advanced gastric cancer, the combination of a 1 L immune checkpoint inhibitor (ICI) and chemotherapy significantly inhibits tumor progression compared with chemotherapy alone [21]. Moreover, cisplatin can induce PD‐L1 expression and increase PD‐L1 expression in a dose‐ and time‐dependent manner in bladder cancer cell lines [22]. In the IMvigor130 trial, tumors with high pretreatment PD‐L1 expression were associated with better outcomes in patients receiving GC [23]. Single‐cell RNA sequencing of circulating immune cells revealed that GC combination therapy upregulates immune‐related transcriptional programs, including those involved in antigen presentation. Combination therapy with nivolumab and GC results in significantly better outcomes in patients with advanced UC than therapy with GC alone, and the combination therapy has been approved as a 1 L chemotherapy in patients with advanced UC by the US Food and Drug Administration [24]. Conversely, the influence of carboplatin on PD‐L1 expression is not well understood, although chemotherapy including carboplatin increases proportions of proliferating CD8 T cells correlated to the immune response in non‐small cell Lung cancer [25]. However, Miyake et al. [7] reported that the best response to 1 L GC, as well as G‐Carbo chemotherapy, is associated with the response to subsequent pembrolizumab treatment. Recently, a French real‐world study, AVENANCE, reported an association between the best response to 1 L chemotherapy and clinical outcomes of maintenance avelumab therapy [26]. In their univariate analyses, patients with PR or SD as the best response to 1 L chemotherapy had a shorter PFS after the induction of avelumab therapy than those with CR as the best response to 1 L chemotherapy. However, the effect of avelumab remains unclear in patients with PR and SD with residual tumors. In the present study, we showed that SD as the best response to 1 L chemotherapy was the only independent prognostic factor associated with shorter PFS and OS after avelumab therapy.

Our results revealed that the median PFS was 7.9 months, estimated OS at 1 year was 77.5%, and ORR was 24.8% in patients with advanced UC who received maintenance avelumab therapy, which were higher than the values in the JAVELIN Bladder 100 trial (3.7 months, 71.3%, and 9.7%, respectively) [2]. In the JAVELIN Bladder 100 trial, EV monotherapy had not yet been approved as a subsequent anticancer therapy for patients with advanced UC that is refractory to prior platinum‐based chemotherapy and ICI, which may be why our results were different from those of JAVELIN Bladder 100 trial. In the AVENANCE study, the median OS from the start of avelumab was 36.0 months in patients who received subsequent EV therapy [26]. Among 73 patients discontinuing avelumab, 36 patients received EV monotherapy as a subsequent anticancer therapy, although 26 patients did not receive a subsequent anticancer drug therapy in our study. It is important that patients who had PD after maintenance avelumab therapy receive EV therapy before they miss an opportunity to receive EV therapy. Moreover, the current study showed that the probabilities of additional 1 year PFS and OS were 77.6% and 93.8%, respectively, in patients who had received 1 year of avelumab therapy. It was recently reported that the probabilities of PFS and OS for an additional year are 66.7% and 93.2%, respectively, in the JAVELIN Bladder 100 trial [27].

Several clinical prognostic risk models have been developed for patients with advanced UC who are treated with chemotherapy and ICI. The well‐known risk model in the pre‐ICI era is the Bellmunt risk score (ECOG‐PS > 0, hemoglobin level < 10 g/dL, and presence of liver metastasis) [5]. Several factors, including serological factors, impaired PS, and metastatic sites, have been reported as prognostic factors in patients with UC who received pembrolizumab [6]. A multicenter retrospective study conducted under the Japan Urological Oncology Group framework proposed a prognostic model based on NLR ≥ 3, hemoglobin < 11 g/dL, metastasis sites (liver, other organs, or lymph nodes only), and ECOG‐PS (≥ 2, 1, or 0) in patients with UC treated with pembrolizumab [28]. Furthermore, five prognostic factors (ECOG‐PS, liver metastasis, platelet count, NLR, and lactate dehydrogenase) have been identified in a clinical trial evaluating atezolizumab, a PD‐L1 inhibitor, following platinum‐based chemotherapy for metastatic UC [29]. The clinicopathological characteristics of chemotherapy responders were similar to those of ICI responders; thus, this may reflect that the response to 1 L chemotherapy was associated with the efficacy of avelumab. Herein, we showed that the CR/PR group had a significantly higher percentage of patients with ECOG‐PS 0 than the SD group and that an ECOG‐PS > 0 was an independent prognostic factor for OS in univariate and multivariate analyses. Among the CR/PR group, 25.0% (17/68) had conditional survival while continuing to use avelumab. Among the 10 patients with liver metastasis, none had received avelumab treatment for ≥ 1 year. Therefore, platinum‐based chemotherapy and maintenance avelumab therapy have limited efficacy for patients with liver metastasis, and other regimens may result in favorable long‐term outcomes.

This study had several limitations. First, it included a limited number of patients and was performed retrospectively, which may have introduced an unknown source of bias. For example, the regimen of 1 L chemotherapy and the number of cycles administered were dependent on each physician. Second, radiographic evaluation intervals depended on institutional protocols and physician discretion. Third, the pathological diagnosis was determined at each institute and not centralized. Fourth, regardless of whether the patient received radical or palliative treatment, a history of radiation therapy was not considered. Radiotherapy of the primary lesion enhances the efficacy of pembrolizumab in patients with advanced UC [30]. Accordingly, additional studies addressing these issues are needed to validate our findings.

In summary, the best response to 1 L chemotherapy may predict clinical outcomes in patients with advanced UC treated with avelumab. In this study, the CR/PR group showed good outcomes with maintenance avelumab therapy. In contrast, the SD group may not expect long‐term survival with maintenance avelumab therapy. Further studies are needed to identify biomarkers to predict the best response to 1 L chemotherapy.

## Author Contributions

**Satoshi Inoue:** conceptualization, data curation, methodology, formal analysis, investigation, project administration, writing – original draft, writing – review and editing. **Akira Hayakawa:** data curation. **Mikinori Kobayashi:** data curation. **Yuriko Nagasaka:** data curation. **Takanao Omi:** data curation. **Noritoshi Shamoto:** data curation. **Fumihiro Ito:** data curation. **Takuma Yuba:** data curation. **Yuri Yuguchi:** data curation. **Hideji Kawanishi:** data curation. **Kosuke Tochigi:** writing – review and editing. **Shusuke Akamatsu:** writing – review and editing, supervision.

## Ethics Statement

This study conformed to the provisions of the Declaration of Helsinki and current ethical guidelines. The entire study was centrally approved by the institutional review board of Nagoya University Graduate School of Medicine (approval number: 2016‐0474‐5) and all hospitals included in this study.

## Consent

The requirement for written informed consent from the participants was waived owing to the retrospective nature of the study.

## Conflicts of Interest

Shusuke Akamatsu is an Editorial Board member of the International Journal of Urology and a co‐author of this article. To minimize bias, he was excluded from all editorial decision‐making related to the acceptance of this article for publication. The other authors declare no conflicts of interest.

## Supporting information


**Figure S1.** Kaplan–Meier curve for time to progression‐free survival (PFS) and overall survival (OS) in patients. PFS (a) and (b) OS (b) of all patients according to the best response to first‐line (1 L) chemotherapy (PR vs. SD).
**Figure S2.** Kaplan–Meier curve for time to overall survival (OS) in patients according to subsequent therapies after maintenance avelumab therapy.

**Table S1.** Characteristics of immune‐related adverse event.

**Table S2.** Baseline clinicopathological characteristics of patients with ≥ 1 year of avelumab.

## Data Availability

The data that support the findings of this study are not openly available owing to reasons of sensitivity and are available from the corresponding author upon reasonable request.

## References

[1] H. von der Maase , L. Sengelov , J. T. Roberts , et al., “Long‐Term Survival Results of a Randomized Trial Comparing Gemcitabine Plus Cisplatin, With Methotrexate, Vinblastine, Doxorubicin, Plus Cisplatin in Patients With Bladder Cancer,” Journal of Clinical Oncology 23 (2005): 4602–4608. 16034041 10.1200/JCO.2005.07.757

[2] T. Powles , S. H. Park , E. Voog , et al., “Avelumab Maintenance Therapy for Advanced or Metastatic Urothelial Carcinoma,” New England Journal of Medicine 383 (2020): 1218–1230. 32945632 10.1056/NEJMoa2002788

[3] T. Powles , B. P. Valderrama , S. Gupta , et al., “Enfortumab Vedotin and Pembrolizumab in Untreated Advanced Urothelial Cancer,” New England Journal of Medicine 390 (2024): 875–888. 38446675 10.1056/NEJMoa2312117

[4] Y. Zhao , X. Xu , Y. Sun , X. Yu , Y. Qi , and X. Dai , “Efficacy and Safety of the First‐Line Systemic Treatments in Patients With Advanced‐Stage Urothelial Carcinoma: A Systematic Review and Network Meta‐Analysis,” Frontiers in Oncology 14 (2024): 1468784. 39351347 10.3389/fonc.2024.1468784 PMC11439625

[5] J. Bellmunt , T. K. Choueiri , R. Fougeray , et al., “Prognostic Factors in Patients With Advanced Transitional Cell Carcinoma of the Urothelial Tract Experiencing Treatment Failure With Platinum‐Containing Regimens,” Journal of Clinical Oncology 28 (2010): 1850–1855. 20231682 10.1200/JCO.2009.25.4599

[6] K. Ito , Y. Kita , and T. Kobayashi , “Real‐World Outcomes of Pembrolizumab for Platinum‐Refractory Advanced Urothelial Carcinoma: Efficacy, Safety, and Evidence for Trial‐Unfit Patients,” International Journal of Urology 30 (2023): 696–703. 36482843 10.1111/iju.15101

[7] M. Miyake , T. Shimizu , N. Nishimura , et al., “Response to Pembrolizumab After Dose‐Reduced Cisplatin Plus Gemcitabine Chemotherapy Is Inferior to That After Carboplatin Plus Gemcitabine Chemotherapy in Cisplatin‐Unfit Patients With Advanced Urothelial Carcinoma,” Clinical Genitourinary Cancer 20 (2022): 196.e191–196.e199. 10.1016/j.clgc.2021.11.006 34916166

[8] National Cancer Institute , “Cancer Therapy Evaluation Program: Protocol Development: Common Terminology Criteria for Adverse Events (CTCAE) v5.0,” https://ctep.cancer.gov/protocolDevelopment/electronic_applications/ctc.htm#ctc_50.

[9] E. A. Eisenhauer , P. Therasse , J. Bogaerts , et al., “New Response Evaluation Criteria in Solid Tumours: Revised RECIST Guideline Version 1.1,” European Journal of Cancer 45 (2009): 228–247. 19097774 10.1016/j.ejca.2008.10.026

[10] T. Kobayashi , K. Ito , T. Kojima , et al., “Pre‐Pembrolizumab Neutrophil‐to‐Lymphocyte Ratio (NLR) Predicts the Efficacy of Second‐Line Pembrolizumab Treatment in Urothelial Cancer Regardless of the Pre‐Chemo NLR,” Cancer Immunology, Immunotherapy 71 (2022): 461–471. 34235546 10.1007/s00262-021-03000-8 PMC10992102

[11] Y. Kanda , “Investigation of the Freely Available Easy‐to‐Use Software ‘EZR’ for Medical Statistics,” Bone Marrow Transplantation 48 (2013): 452–458. 23208313 10.1038/bmt.2012.244 PMC3590441

[12] L. Galluzzi , A. Buqué , O. Kepp , L. Zitvogel , and G. Kroemer , “Immunogenic Cell Death in Cancer and Infectious Disease,” Nature Reviews. Immunology 17 (2017): 97–111. 10.1038/nri.2016.107 27748397

[13] L. Zitvogel , L. Apetoh , F. Ghiringhelli , and G. Kroemer , “Immunological Aspects of Cancer Chemotherapy,” Nature Reviews. Immunology 8 (2008): 59–73. 10.1038/nri2216 18097448

[14] C. Rébé , L. Demontoux , T. Pilot , and F. Ghiringhelli , “Platinum Derivatives Effects on Anticancer Immune Response,” Biomolecules 10 (2019): 13. 31861811 10.3390/biom10010013 PMC7022223

[15] A. R. de Biasi , J. Villena‐Vargas , and P. S. Adusumilli , “Cisplatin‐Induced Antitumor Immunomodulation: A Review of Preclinical and Clinical Evidence,” Clinical Cancer Research 20 (2014): 5384–5391. 25204552 10.1158/1078-0432.CCR-14-1298 PMC4216745

[16] S. Wan , S. Pestka , R. G. Jubin , Y. L. Lyu , Y. C. Tsai , and L. F. Liu , “Chemotherapeutics and Radiation Stimulate MHC Class I Expression Through Elevated Interferon‐Beta Signaling in Breast Cancer Cells,” PLoS One 7 (2012): e32542. 22396773 10.1371/journal.pone.0032542 PMC3291570

[17] S. V. Hato , A. Khong , I. J. M. de Vries , and W. J. Lesterhuis , “Molecular Pathways: The Immunogenic Effects of Platinum‐Based Chemotherapeutics,” Clinical Cancer Research 20 (2014): 2831–2837. 24879823 10.1158/1078-0432.CCR-13-3141

[18] S. J. Park , W. Ye , R. Xiao , et al., “Cisplatin and Oxaliplatin Induce Similar Immunogenic Changes in Preclinical Models of Head and Neck Cancer,” Oral Oncology 95 (2019): 127–135. 31345380 10.1016/j.oraloncology.2019.06.016 PMC6662630

[19] L. Fournel , Z. Wu , N. Stadler , et al., “Cisplatin Increases PD‐L1 Expression and Optimizes Immune Check‐Point Blockade in Non‐Small Cell Lung Cancer,” Cancer Letters 464 (2019): 5–14. 31404614 10.1016/j.canlet.2019.08.005

[20] C. L. Chang , Y. T. Hsu , C. C. Wu , et al., “Dose‐Dense Chemotherapy Improves Mechanisms of Antitumor Immune Response,” Cancer Research 73 (2013): 119–127. 23108141 10.1158/0008-5472.CAN-12-2225 PMC3537885

[21] Q. Xu , D. Yi , C. Jia , F. Kong , and Y. Jia , “Immunotherapy Plus Chemotherapy Versus Chemotherapy Alone in the First‐Line Treatment for Advanced Gastric Cancer/Gastroesophageal Junction Cancer: A Real‐World Retrospective Study,” Frontiers in Immunology 15 (2024): 1463017. 39575245 10.3389/fimmu.2024.1463017 PMC11578984

[22] T. F. Tsai , J. F. Lin , Y. C. Lin , et al., “Cisplatin Contributes to Programmed Death‐Ligand 1 Expression in Bladder Cancer Through ERK1/2‐AP‐1 Signaling Pathway,” Bioscience Reports 39 (2019): BSR20190362. 31341011 10.1042/BSR20190362 PMC6783655

[23] M. D. Galsky , X. Guan , R. Banchereau , et al., “658MO Cisplatin (Cis)‐Related Immunomodulation and Efficacy With Atezolizumab (Atezo) + Cis‐ vs Carboplatin (Carbo)‐Based Chemotherapy (Chemo) in Metastatic Urothelial Cancer (mUC),” Annals of Oncology 32 (2021): S682–S683.

[24] M. S. van der Heijden , G. Sonpavde , T. Powles , et al., “Nivolumab Plus Gemcitabine‐Cisplatin in Advanced Urothelial Carcinoma,” New England Journal of Medicine 389 (2023): 1778–1789. 37870949 10.1056/NEJMoa2309863 PMC12314471

[25] P. L. de Goeje , M. Poncin , K. Bezemer , et al., “Induction of Peripheral Effector CD8 T‐Cell Proliferation by Combination of Paclitaxel, Carboplatin, and Bevacizumab in Non‐Small Cell Lung Cancer Patients,” Clinical Cancer Research 25 (2019): 2219–2227. 30642911 10.1158/1078-0432.CCR-18-2243

[26] P. Barthélémy , C. Thibault , A. Fléchon , et al., “Real‐World Study of Avelumab First‐Line Maintenance Treatment in Patients With Advanced Urothelial Carcinoma in France: Overall Results From the Noninterventional AVENANCE Study and Analysis of Outcomes by Second‐Line Treatment,” European Urology Oncology 8 (2025): 407–416. 39448350 10.1016/j.euo.2024.09.014

[27] P. Grivas , S. H. Park , E. Voog , et al., “1975P Avelumab First‐Line (1L) Maintenance in Advanced Urothelial Carcinoma (aUC): Conditional Survival and Long‐Term Safety in Patients (Pts) Treated for ≥ 1 or ≥ 2 Years in JAVELIN Bladder 100,” Annals of Oncology 35 (2024): S1144.

[28] T. Kobayashi , K. Ito , T. Kojima , et al., “Risk Stratification for the Prognosis of Patients With Chemoresistant Urothelial Cancer Treated With Pembrolizumab,” Cancer Science 112 (2021): 760–773. 33283385 10.1111/cas.14762 PMC7893997

[29] G. Sonpavde , J. Manitz , C. Gao , et al., “Five‐Factor Prognostic Model for Survival of Post‐Platinum Patients With Metastatic Urothelial Carcinoma Receiving PD‐L1 Inhibitors,” Journal of Urology 204 (2020): 1173–1179. 32552295 10.1097/JU.0000000000001199 PMC7655635

[30] H. Fukushima , T. Kijima , S. Fukuda , et al., “Impact of Radiotherapy to the Primary Tumor on the Efficacy of Pembrolizumab for Patients With Advanced Urothelial Cancer: A Preliminary Study,” Cancer Medicine 9 (2020): 8355–8363. 32886446 10.1002/cam4.3445 PMC7666746

