# Supplementary material for: Response to First‐Line Chemotherapy Predicts Response to Maintenance Avelumab Therapy in Japanese Patients With Advanced Urothelial Carcinoma
Source: Int J Urol. 2025 Jul 2;32(10):1449–59. doi: 10.1111/iju.70162 (PMC12503202; doi:10.1111/iju.70162)
Supplement: Supplementary file 1 — Figure S1. Kaplan–Meier curve for time to progression‐free survival (PFS) and overall survival (OS) in patients. PFS (a) and (b) OS (b) of all patients according to the best response to first‐line (1 L) chemotherapy (PR vs. SD). Figure S2. Kaplan–Meier curve for time to overall survival (OS) in patients according to subsequent therapies after maintenance avelumab therapy. [file IJU-32-1449-s001.docx]

**Abbreviations & Acronyms**

PR = partial response

SD = stable disease


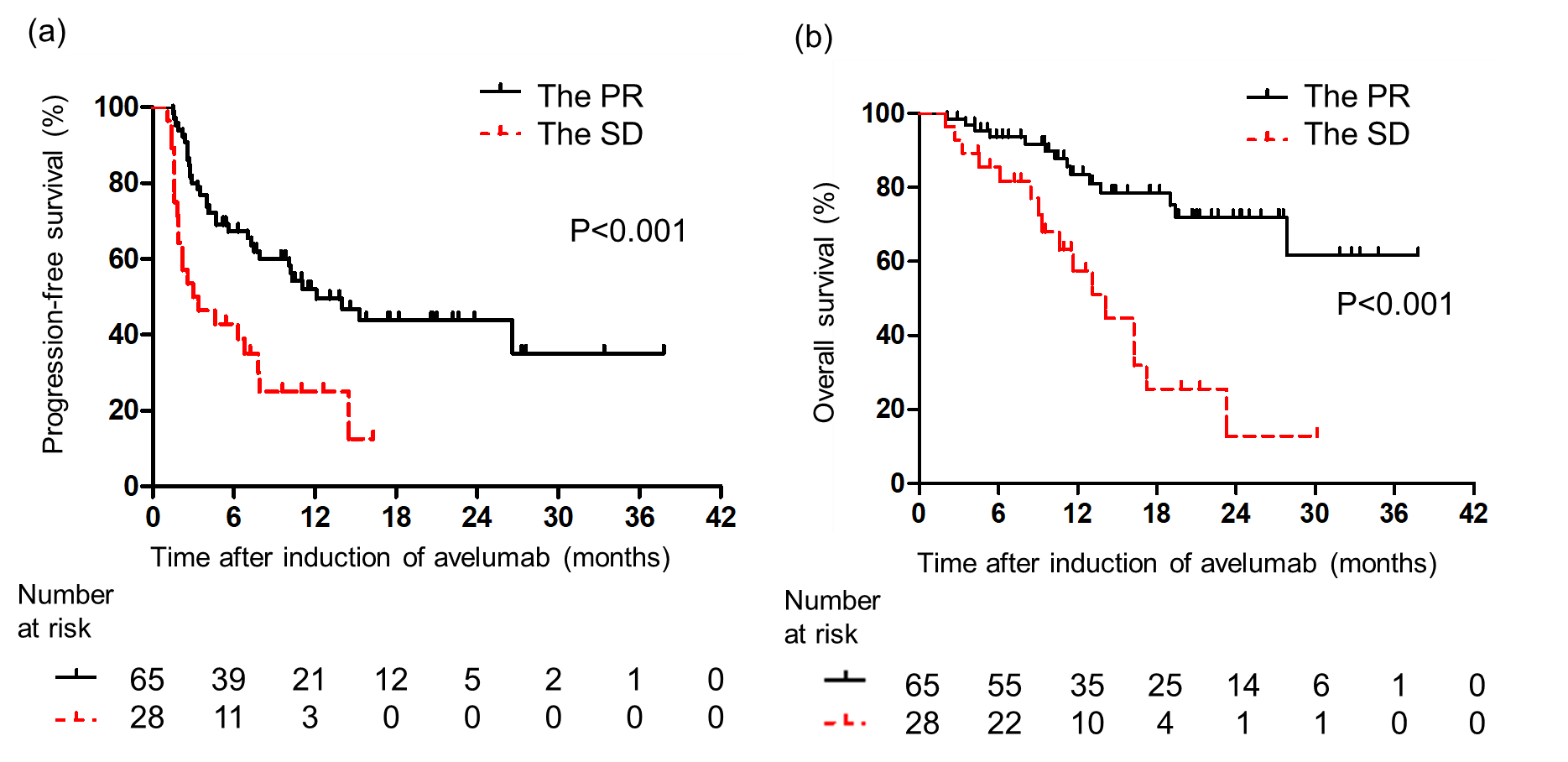


**FIGURE S1** Kaplan-Meier curve for time to progression-free survival (PFS) and overall survival (OS) in patients. PFS (a) and (b) OS (b) of all patients according to the best response to first-line (1L) chemotherapy (PR vs SD)


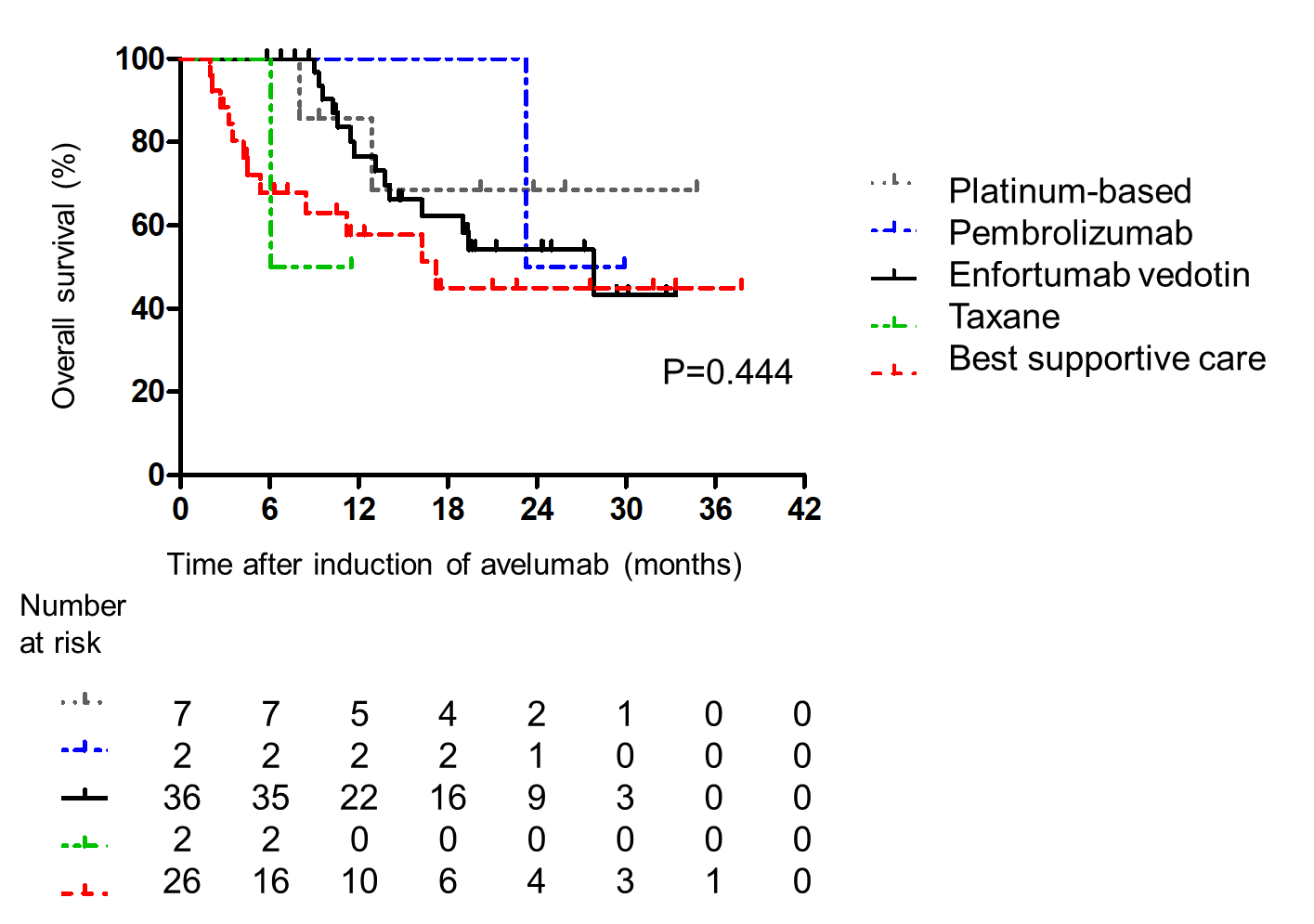


**FIGURE S2** Kaplan-Meier curve for time to overall survival (OS) in patients according to subsequent therapies after maintenance avelumab therapy.
