# Supplementary material for: Response to First‐Line Chemotherapy Predicts Response to Maintenance Avelumab Therapy in Japanese Patients With Advanced Urothelial Carcinoma
Source: Int J Urol. 2025 Jul 2;32(10):1449–59. doi: 10.1111/iju.70162 (PMC12503202; doi:10.1111/iju.70162)
Supplement: Supplementary file 2 — Table S1. Characteristics of immune‐related adverse event. [file IJU-32-1449-s003.docx]

Table S1. Characteristics of immune-related adverse event

| Event | Any grade, n (%) | Grade 3 or more, n (%) |
| --- | --- | --- |
| Any event | 34 (34.0) | 9 (9.0) |
|  |  |  |
| Rash | 9 (9.0) | 1 (1.0) |
| Infusion-related reaction | 8 (8.0) | 0 (0.0) |
| Interstitial lung disease | 5 (5.0) | 1 (1.0) |
| Adrenal insufficiency | 4 (4.0) | 4 (4.0) |
| Hypothyroidism | 3 (3.0) | 0 (0.0) |
| Colitis | 3 (3.0) | 1 (1.0) |
| Myositis | 2 (2.0) | 1 (1.0) |
| Renal dysfunction | 2 (2.0) | 1 (1.0) |
| Type 1 diabetes mellitus | 1 (1.0) | 0 (0.0) |
| Hyperthyroidism | 1 (1.0) | 0 (0.0) |
| Dysphagia | 1 (1.0) | 0 (0.0) |
| Liver dysfunction | 1 (1.0) | 1 (1.0) |
