# Supplementary material for: Response to First‐Line Chemotherapy Predicts Response to Maintenance Avelumab Therapy in Japanese Patients With Advanced Urothelial Carcinoma
Source: Int J Urol. 2025 Jul 2;32(10):1449–59. doi: 10.1111/iju.70162 (PMC12503202; doi:10.1111/iju.70162)
Supplement: Supplementary file 3 — Table S2. Baseline clinicopathological characteristics of patients with ≥ 1 year of avelumab. [file IJU-32-1449-s002.docx]

**Abbreviations & Acronyms**

CR = complete response

ddMVAC = dose-dense methotrexate, vinblastine, doxorubicin, and cisplatin

ECOG-PS = Eastern Cooperative Oncology Group performance status

GC = gemcitabine and cisplatin

G-Carbo = Gemcitabine and carboplatin

irAEs = immune-related adverse events

NLR = neutrophil-to-lymphocyte ratio

PR = partial response

SD = stable disease

UC = urothelial carcinoma

Table S2. Baseline clinicopathological characteristics of patients with ≥1 year of avelumab

|  |  |  |  | First-line chemotherapy | | |  |  |
| --- | --- | --- | --- | --- | --- | --- | --- | --- |
| Variables |  | Total |  | The CR/PR |  | The SD |  | P-value |
|  |  | n=19 |  | n=17 |  | n=2 |  |  |
| Age (year) |  |  |  |  |  |  |  | 0.110 |
|  | Median | 74 |  | 75 |  | 62.5 |  |  |
|  | Range | 55-83 |  | 64-83 |  | 55-70 |  |  |
| Follow-up duration (month) |  |  |  |  |  |  |  | 0.054 |
|  | Median | 22.6 |  | 23.0 |  | 14.9 |  |  |
|  | Range | 12.6-37.8 |  | 14.6-37.8 |  | 12.6-17.2 |  |  |
| Cycles of avelumab |  |  |  |  |  |  |  | 0.163 |
|  | Median | 37 |  | 37 |  | 23.5 |  |  |
|  | Range | 19-59 |  | 19-59 |  | 21-26 |  |  |
| Sex, n (%) |  |  |  |  |  |  |  | 1.000 |
|  | Male | 16 (84.2) |  | 3 (17.6) |  | 0 (0.0) |  |  |
|  | Female | 3 (15.8) |  | 14 (82.4) |  | 2 (100.0) |  |  |
| ECOG-PS, n (%) |  |  |  |  |  |  |  | 0.468 |
|  | 0 | 14 (73.7) |  | 13 (76.5) |  | 1 (50.0) |  |  |
|  | 1 | 5 (26.3) |  | 4 (23.5) |  | 1 (50.0) |  |  |
|  | 2-4 | 0 (0.0) |  | 0 (0.0) |  | 0 (0.0) |  |  |
| Histology, n (%) |  |  |  |  |  |  |  | 1.000 |
|  | Pure UC | 16 (84.2) |  | 14 (82.4) |  | 2 (100.0) |  |  |
|  | UC with divergent differentiation or histological subtype | 3 (15.8) |  | 3(17.6) |  | 0 (0.0) |  |  |
| Primary site, n (%) |  |  |  |  |  |  |  | 0.550 |
|  | Upper urinary tract | 7 (36.8) |  | 7 (41.2) |  | 0 (0.0) |  |  |
|  | Bladder | 11 (57.9) |  | 9 (52.9) |  | 2 (100.0) |  |  |
|  | Both | 1 (5.3) |  | 1 (5.9) |  | 0 (0.0) |  |  |
| Hydronephrosis, n (%) |  |  |  |  |  |  |  | 1.000 |
|  | Yes | 4 (21.1) |  | 13 (76.5) |  | 2 (100.0) |  |  |
|  | No | 15 (78.9) |  | 4 (23.5) |  | 0 (0.0) |  |  |
| Smoking status, n (%) |  |  |  |  |  |  |  | 1.000 |
|  | Never smoker | 4 (21.1) |  | 4 (23.5) |  | 0 (0.0) |  |  |
|  | Former smoker | 11 (57.9) |  | 9 (52.9) |  | 2 (100.0) |  |  |
|  | Current smoker | 3 (15.8) |  | 3 (17.6) |  | 0 (0.0) |  |  |
|  | Unknown | 1 (5.3) |  | 1 (5.9) |  | 0 (0.0) |  |  |
| Metastatic or locally advanced disease, n (%) |  |  |  |  |  |  |  | - |
|  | Metastatic | 19 (100.0) |  | 17 (100.0) |  | 2 (100.0) |  |  |
|  | Locally advanced | 0 (0.0) |  | 0 (0.0) |  | 0 (0.0) |  |  |
| Surgical removal of primary organ, n (%) |  |  |  |  |  |  |  | 0.509 |
|  | Yes | 12 (63.2) |  | 10 (58.8) |  | 0 (0.0) |  |  |
|  | No | 7 (38.8) |  | 7 (41.2) |  | 2 (100.0) |  |  |
| Neoadjuvant chemotherapy, n (%) |  |  |  |  |  |  |  | 0.018 |
|  | Yes | 3 (15.8) |  | 1 (5.9) |  | 2 (100.0) |  |  |
|  | No | 16 (84.2) |  | 16 (94.1) |  | 0 (0.0) |  |  |
| Adjuvant chemotherapy, n (%) |  |  |  |  |  |  |  | 1.000 |
|  | Yes | 1 (5.3) |  | 1 (5.9) |  | 0 (0.0) |  |  |
|  | No | 18 (94.7) |  | 16 (94.1) |  | 2 (100.0) |  |  |
| Hemoglobin concentration <10 g/dL, n (%) |  |  |  |  |  |  |  | 0.485 |
|  | Yes | 8 (42.1) |  | 8 (47.1) |  | 0 (0.0) |  |  |
|  | No | 11 (57.9) |  | 9 (52.9) |  | 2 (100.0) |  |  |
| NLR >3, n (%) |  |  |  |  |  |  |  | 0.468 |
|  | Yes | 5 (26.3) |  | 4 (23.5) |  | 1 (50.0) |  |  |
|  | No | 14 (73.7) |  | 13 (76.5) |  | 1 (50.0) |  |  |
| Lymph node-only metastasis, n (%) |  |  |  |  |  |  |  | 1.000 |
|  | Yes | 10 (52.6) |  | 9 (52.9) |  | 1 (50.0) |  |  |
|  | No | 9 (47.4) |  | 8 (47.1) |  | 1 (50.0) |  |  |
| Visceral metastasis, n (%) |  |  |  |  |  |  |  | 1.000 |
|  | Yes | 7 (36.8) |  | 6 (35.3) |  | 1 (50.0) |  |  |
|  | No | 12 (63.2) |  | 11 (64.7) |  | 1 (50.0) |  |  |
| Liver metastasis, n (%) |  |  |  |  |  |  |  | - |
|  | Yes | 0 (0.0) |  | 0 (0.0) |  | 0 (0.0) |  |  |
|  | No | 19 (100.0) |  | 17 (100.0) |  | 2 (100.0) |  |  |
| Bellmunt risk factors*, n(%) |  |  |  |  |  |  |  | 1.000 |
|  | 0 | 9 (47.4) |  | 8 (47.1) |  | 1 (50.0) |  |  |
|  | 1 | 7 (36.8) |  | 6 (35.3) |  | 1 (50.0) |  |  |
|  | 2 | 3 (15.8) |  | 3 (17.6) |  | 0 (0.0) |  |  |
|  | 3 | 0 (0.0) |  | 0 (0.0) |  | 0 (0.0) |  |  |
| First-line chemotherapy regimen, n (%) |  |  |  |  |  |  |  | 0.105 |
|  | GC | 9 (47.4) |  | 8 (47.1) |  | 1 (50.0) |  |  |
|  | G-Carbo | 9 (47.4) |  | 9 (52.9) |  | 0 (0.0) |  |  |
|  | ddMVAC | 1 (5.3) |  | 0 (0.0) |  | 1 (50.0) |  |  |
|  | GC or G-Carbo^†^ | 0 (0.0) |  | 0 (0.0) |  | 0 (0.0) |  |  |
| Cycles of first-line chemotherapy, n (%) |  |  |  |  |  |  |  | 1.000 |
|  | <4 | 4 (21.1) |  | 4 (23.5) |  | 0 (0.0) |  |  |
|  | 4 | 14 (73.7) |  | 12 (70.6) |  | 2 (100.0) |  |  |
|  | 5-6 | 1 (5.3) |  | 1 (5.9) |  | 0 (0.0) |  |  |
| Subsequent anticancer therapy, n (%) |  |  |  |  |  |  |  | 1.000 |
|  | Platinum-based | 1 (5.3) |  | 1 (5.9) |  | 0 (0.0) |  |  |
|  | Pembrolizumab | 0 (0.0) |  | 0 (0.0) |  | 0 (0.0) |  |  |
|  | Enfortumab vedotin | 2 (10.5) |  | 2 (11.8) |  | 0 (0.0) |  |  |
|  | Taxane-based | 0 (0.0) |  | 0 (0.0) |  | 0 (0.0) |  |  |
|  | Best supportive care | 5 (26.3) |  | 4 (23.5) |  | 1 (50.0) |  |  |
|  | Continuing avelumab | 11 (57.9) |  | 10 (58.8) |  | 1 (50.0) |  |  |
| IrAE, n (%) |  |  |  |  |  |  |  | 1.000 |
|  | Yes | 9 (47.4) |  | 9 (52.9) |  | 1 (50.0) |  |  |
|  | No | 10 (52.6) |  | 8 (47.1) |  | 1 (50.0) |  |  |

*Bellmunt risk factors include an ECOG-PS score >0, a hemoglobin concentration of <10 g/dL, and the presence of liver metastases. †This category includes patients who switched platinum-based regimens while receiving first-line chemotherapy.
